# Primary care and youth mental health in Ireland: qualitative study in deprived urban areas

**DOI:** 10.1186/1471-2296-14-194

**Published:** 2013-12-17

**Authors:** Dorothy Leahy, Elisabeth Schaffalitzky, Claire Armstrong, Gerard Bury, Paula Cussen-Murphy, Rachel Davis, Barbara Dooley, Blanaid Gavin, Rory Keane, Eamon Keenan, Linda Latham, David Meagher, Pat McGorry, Fiona McNicholas, Ray O’Connor, Ellen O’Dea, Veronica O’Keane, Tom P O’Toole, Edel Reilly, Patrick Ryan, Lena Sanci, Bobby P Smyth, Walter Cullen

**Affiliations:** 1 Graduate Entry Medical School, University of Limerick, Limerick, Ireland; 2 UCD School of Medicine & Medical Science, University College Dublin, Belfield, Dublin 4, Ireland; 3 HSE Mid-West, Limerick, Ireland; 4 Child & Adolescent Mental Health Services, HSE Mid-West, Limerick, Ireland; 5 School of Psychology, University College Dublin, Belfield, Dublin 4, Ireland; 6 Department of Child & Adolescent Psychiatry, Lucena Clinic, Rathgar, Dublin 6, Ireland; 7 Sláinte Drug & Alcohol Services, HSE Mid-West, Limerick, Ireland; 8 Addiction Services, HSE Dublin Mid-Leinster, Dublin, Ireland; 9 Thomas Court Primary Care Centre, Dublin 8, Ireland; 10 Orygen Youth Health Research Centre, University of Melbourne, Melbourne, Australia; 11 HSE Dublin Mid-Leinster, Dublin, Ireland; 12 Trinity Centre for Health Sciences, Tallaght Hospital, Dublin 24, Ireland; 13 Providence VA Medical Centre & Warren Alpert Medical School, Brown University, Rhode Island, USA; 14 Fatima Regeneration Board, Rialto, Dublin 8, Ireland; 15 Department of Psychology, University of Limerick, Limerick, Ireland; 16 Department of General Practice, University of Melbourne, Melbourne, Australia; 17 Department of Public Health & Primary Care, Trinity College Dublin, Ireland

**Keywords:** Young people, Urban deprivation, Mental health, Substance use, Primary care, General practice

## Abstract

**Background:**

Mental disorders account for six of the 20 leading causes of disability worldwide with a very high prevalence of psychiatric morbidity in youth aged 15–24 years. However, healthcare professionals are faced with many challenges in the identification and treatment of mental and substance use disorders in young people (e.g. young people’s unwillingness to seek help from healthcare professionals, lack of training, limited resources etc.) The challenge of youth mental health for primary care is especially evident in urban deprived areas, where rates of and risk factors for mental health problems are especially common. There is an emerging consensus that primary care is well placed to address mental and substance use disorders in young people especially in deprived urban areas. This study aims to describe healthcare professionals’ experience and attitudes towards screening and early intervention for mental and substance use disorders among young people (16–25 years) in primary care in deprived urban settings in Ireland.

**Methods:**

The chosen method for this qualitative study was inductive thematic analysis which involved semi-structured interviews with 37 healthcare professionals from primary care, secondary care and community agencies at two deprived urban centres.

**Results:**

We identified three themes in respect of interventions to increase screening and treatment: (1) Identification is optimised by a range of strategies, including raising awareness, training, more systematic and formalised assessment, and youth-friendly practices (e.g. communication skills, ensuring confidentiality); (2) Treatment is enhanced by closer inter-agency collaboration and training for all healthcare professionals working in primary care; (3) Ongoing engagement is enhanced by motivational work with young people, setting achievable treatment goals, supporting transition between child and adult mental health services and recognising primary care’s longitudinal nature as a key asset in promoting treatment engagement.

**Conclusions:**

Especially in deprived areas, primary care is central to early intervention for youth mental health. Identification, treatment and continuing engagement are likely to be enhanced by a range of strategies with young people, healthcare professionals and systems. Further research on youth mental health and primary care, including qualitative accounts of young people’s experience and developing complex interventions that promote early intervention are priorities. (350 words)

## Background

Mental and substance use disorders contribute the largest disease burden in young people [1,2], with three-quarters first emerging between the ages of fifteen and twenty five [3]. In Ireland, psychological morbidity has been reported in 21-27% of young adults [4], while the rate of youth suicide is the fourth highest of 26 European Union countries [5]. Young people attend primary care regularly and as they often present with coexisting risk behaviour / psychosocial problems, primary care is ideally placed to address these issues opportunistically [6]. However, healthcare professionals face many challenges when trying to identify youth mental and substance use disorders, including interpreting the developmental changes that coincide with adolescence as a mental disorder [1], fear of ‘over-medicalising’ young lives and misinterpreting depression as a normal response to the wider psychosocial context of a young person’s life [7].

Many health professionals, including GPs may not be entirely comfortable with identifying / treating young people with emotional mental / substance use disorders. Previous research found that an exploration of psychological issues does not always take place in GP consultations, even when the doctor feels that these are present and the adolescent is similarly aware [8,9]. In the US, the median rate of recognition of youth mental health problems by GPs was only 18%, and was often initiated as a result of parental concerns [10]. Findings from Fleury and colleagues (2012) suggested that GPs rarely used clinical screening tools or collaborated with other healthcare professionals, and tended to limit treatment options to monitoring medication or providing support therapy [11]. Other studies have found that most primary care clinicians do not routinely screen for suicide risk [9,12] and nearly 60% of youth in need of mental health services do not receive the care they need, even after suicide attempt [13].

Lack of time and training are often mentioned by healthcare professionals in primary care as major barriers to a comprehensive psychosocial diagnosis [14]–[16]. Other barriers included lack of financial reimbursement for uncompensated time spent on mental health screening [17], limited knowledge about suicide risk, poor availability of mental health services for referral [18], insufficient time to discuss mental health problems during consultations, restricted resources for screening (e.g. space, computers and staff) [12], patient confidentiality issues [9], lack of clearly defined guidelines, ineffective communication skills and reluctance to discuss sensitive issues [19] and in some cases healthcare professionals own stigmatising attitudes towards mental illness [20]. Additionally young people themselves may be reluctant to contact healthcare professionals, or even recognise them as a source of help when distressed [7].

The challenge of youth mental health for healthcare professionals is especially evident in socio-economically disadvantaged areas where risk factors for mental health problems are especially common [21]–[23], in addition to associated adverse psychosocial outcomes, like homelessness and drug use [24]–[26]. This concentration of health and social problems creates a level of demand which places substantial and continuous pressures on healthcare professionals [27].

There is a dearth of evidence regarding the experiences of and attitudes towards screening and treatment of mental and substance use disorders among healthcare professionals and young people in the Irish healthcare system and to date no clinical guidelines in relation to screening and early intervention have been published. However a similar approach has been employed in the development of clinical guidelines to inform hepatitis C management among current or former injecting drug users [28,29]. In order to create future interventions for this population, it is important to understand how current practice with youth mental health in urban deprived areas is experienced by those who work within it: This knowledge ensures interventions will be tailored to the context and will address the relevant domains for improved services and outcomes. Since 2011, our study group has been working towards developing an intervention which addresses barriers to ‘early intervention’ for mental and substance use disorders that is evidence based, feasible and acceptable to young people and healthcare professionals. This work includes three phases:

*Phase 1*: will describe the experience of (and attitudes towards) screening and early intervention for mental / substance use disorders by interviewing a purposive sample of young people / healthcare professionals recruited from community agencies and primary / secondary care.

*Phase 2*: will develop a ‘complex intervention’ to improve screening and early intervention that is informed by the findings of phase 1, scientific evidence and a Delphi-facilitated expert consensus process.

*Phase 3*: will provide iterative feedback to participating healthcare professionals in the study from phase 1, 2 and determine what if any care components have been incorporated and any barriers encountered.

The current paper is based on findings from the first phase of the study, where one of the key aims was to describe healthcare professionals’ experience of (and attitudes towards) screening and early intervention for mental and substance use disorders among young people in primary care in deprived urban settings. Definitions of youth in the current study are in line with previous work on youth mental health where the terms ‘youth’ or ‘young people’ are often used to describe people within the 12 to 25 age range [6,30].

## Methods

### Overview

A qualitative study employing semi-structured interviews with 37 healthcare professionals working with young people at a diverse range of health and social care agencies in two deprived urban centres.

### Body giving ethics approval

The project was reviewed / approved by the following research ethics committees: Irish College of General Practitioners, St James’s / Federated Dublin Voluntary Hospitals, Lucena / St John of Gods, HSE - Midwest Regional Hospital.

### Setting

The study took place in Limerick and Dublin South Inner City during 2011–12. Both centres contain some of Ireland’s most deprived local areas, with 15 of Limerick city’s 37 electoral divisions receiving deprivation scores in the disadvantaged or extremely disadvantaged categories [31]. As a result, youth mental health [32,33] and problem drug use are priority issues [34]. In Dublin South Inner City, this has been the case for over thirty years [25].

### Sampling and recruitment

Based on previous research examining similar questions among similar populations [35,36], we estimated 16–32 health / social care professionals would need to be interviewed to reach theoretical saturation. The study sample were health and social care professionals at agencies and practices which interact with primary care, and reflect the range of settings where young people seek help for mental and substance use disorders. We adopted a purposive sampling framework, which included geographical region and health / social care agency as sampling parameters. The conceptual framework for this study is based on two well established and studied theoretic models: (1) ‘*Social Determinants of Health*’, which emphasises the role of social deprivation and social cohesion in the effective treatment of mental illnesses [21]. Thus, the current study’s placement in areas of urban deprivation (in Dublin and Limerick) where GP and primary care sites have the potential to liaise with community resources to address youth mental health, reflect this relationship.

The (2)‘*Chronic Care Model*’, describes how six interdependent facets of primary care delivery (*self*-*management support*, *clinical information systems*, *delivery system redesign*, *decision support*, *health care organisation*, *and community resources*) can effectively improve patient satisfaction and chronic disease outcome measures in a variety of health care settings including low income communities [37]. Mental illness is applicable to the Chronic Care Model in terms of its chronicity, need for monitoring, care adjustments, and multifaceted interventions. Study settings included primary care itself (general practices, primary care teams), secondary care (adult mental health services, child and adolescent mental health services, addiction services) and community agencies. Healthcare professionals at each participating site were identified by a member of the Project Steering Group and invited to participate. In total, 37 health professionals were interviewed (see Table 1 for breakdown of occupation and healthcare sector).

**Table 1:** Demographic information for study participants

**Demographic information**	**Number of sample**	**Percentage of sample**
*Gender*		
• Male	12	32.4
• Female	25	67.5
*Number of years in current post*		
• <1 year	2	5.4
• 1-5 years	17	45.9
• >5 years	18	48.6
*Healthcare Sector*		
• Primary Care	13	35.1
• Secondary Care		
o Mental Health Services	9	24.3
o Addiction Services	3	8.1
• Community Agencies	12	32.4
*Professional Background*		
• Addiction (outreach / counselling)	6	16.2
• Counselling / psychology	2	5.4
• Extern / Youth Workers	8	21.6
• Medical (GPs / Psychiatrists)	9	24.3
• Nursing	8	21.6
• Primary Care other (e.g. social work, speech & language therapy)	4	10.8
**Total Sample**	**37**	**100.0**

### Data collection

Interviews elicited information on participants’ experience of mental and substance use disorders among young adults and attitudes towards screening and early intervention. The interview topic guide (see Appendix 1) was informed by a literature review on the role of general practice in addressing youth mental health [38], and theoretic frameworks, Social Determinants of Health [21] and the ‘Chronic Care Model [37]. Interviews were conducted in person, in a quiet room, ranged in length from 16–120 minutes, and were digitally recorded.

The topic guide served as a guiding framework for the interview rather than a prescriptive line of questioning, thus every effort was made to allow participants to elaborate on aspects of key importance to them [39]. All of the participants were interviewed in their work place, thus in some cases the interview duration was dependent on external factors related to the participant’s work environment. Interview material was reviewed after every 2–3 interviews to allow researchers identify new issues to explore in subsequent interviews and to note emerging / diverging consensus.

### Data analysis

Data were analysed using an inductive thematic approach to analysis in accordance with Braun and Clarke’s five-step process e.g. 1) Familiarisation with the data; 2) Generating initial codes; 3) Searching for the themes; 4) Reviewing the themes; 5) Defining and naming themes [40]. Thus, each transcript was transcribed verbatim and reviewed by the interviewers for accuracy. Interviews were openly coded to allow concept categories to occur without prior assumptions. The researchers analysed interview transcripts in groups which were representative of the various study settings e.g. (addiction clinics, mental health services, general practices etc.) to identify common codes for each group. Transcripts were read repeatedly and constant collaboration used to ensure codes created were accurately reflective of the data. Transcript data was entered into the qualitative research package NVivo 9. As transcripts were coded new codes emerged and were added to the coding list. Coded information was sorted into categories and themes were identified from these categories. Three researchers (DL, ES, CA) coded the interviews individually and corroborated themes with the principal investigator (WC) to reach inter-rater reliability. All researchers had access to coding materials and followed an agreed coding protocol where any new codes, and changes to existing codes were highlighted as advised by Boyatzis [41]. Findings were compared with other study findings for the purposes of validity and reliability. A narrative analytic account, supported by verbatim extracts from each participant, was developed.

## Results

Two Meta / key overarching themes emerged from the healthcare professional data: 1) The role of *Context* in screening and treating youth mental and substance use disorders and 2) *Intervention* and the associated barriers and enablers with respect to screening and treatment. Of direct relevance to this paper on healthcare professional perspectives, is the *Intervention* theme which comprised three distinct subthemes: (i) *Identification*, (ii) *Treatment*, and (iii) *On*-*going engagement*. Each of these subthemes was further disaggregated into the barriers to and enablers of each process (see Table 2).

**Table 2:** Barriers to and enablers of identification**,** treatment and on**-**going engagement

***Theme***	***Enabler***	***Barrier***
*Identification of mental health and*/*or substance use problem in a young person*	• Outreach work	• Prioritisation of crisis cases at the expense of early intervention
	• Activity-based engagement	• Confidentiality and consent issues, particularly around parental involvement for under 18s
	• Mental health/drug awareness promotion	• Concerns around formally treating a young person for a mental health or substance use problem
	• Specific training in youth mental health/substance use problems	
	• Experience in dealing with young people with mental health/substance use problems	
	• Using formal assessment tools	
	• Building a trusting relationship with the young person	
*Treatment of young person for mental health and*/*or substance use problem*	• A holistic/collaborative approach, including high quality communication, between healthcare agencies, e.g. primary care, secondary care and community-based agencies	• Limited funding resources result in a lack of age-appropriate services
	• Training for primary healthcare professionals, in particular, GPs in addressing youth mental health and substance use problems effectively.	• Crisis intervention taking precedence over early intervention
*On*-*going engagement*	• Intrinsic motivation of the young person	• If young person is attending because of external pressure
	• Continued/repeated opportunities for engagement	• Unwillingness of some young people to attend counselling
	• Personal achievement goals	• Transition from child to adult services
	• Having the structure of school or work to continue with during treatment or return to after treatment	

### Theme 1: Identification – Barriers

#### ‘Prioritisation of crisis cases’

Healthcare professionals described feeling “over whelmed” and “stretched” when discussing the provision of screening services for young people. The most commonly reported barriers to identifying mental and substance use disorders in a young person for healthcare professionals, related to care of acutely unwell young people having to take precedence over those with less acute or severe problems. Thus some healthcare professionals felt that there was “a huge gap” in services for young people with less severe mental health problems or those in the initial stages of a mental illness who would benefit from early intervention.

“*The people who end up getting referred to mental health services are the tip of an absolutely enormous iceberg. It is one in a hundred. It is tiny and it is getting tinier all the time. Their criteria for who they will see*, *which in a way*, *I can understand but it still leaves this huge gap*” (GP).

#### ‘Confidentiality and consent issues’

Healthcare professionals also felt restricted due to confidentiality and consent issues and described them as a major barrier to the identification of mental and substance use issues in young people, particularly when this related to parental involvement for those aged under 18. Treating a young person for (and fear of labelling them with) a substance use problem was also cited as an important barrier to identification because of the associated potential long term implications of such a diagnosis.

“*Occupation wise and college wise*…*they usually ask the GP for medical records. The GP will have our letters on file so realistically*, *if they are dealing with a substance abuse problem I try and keep it separate*, *because then there is less information on their file that would prevent them getting a place or a job*” (Psychiatrist).

#### ‘Concerns around formally treating a young person’

Variable access to community-based, non-pharmacological interventions, adopting a ‘watchful waiting’ approach to management and misattributing mental and substance use disorders to developmental changes were additional mechanisms that can delay identification.

“*So there*’*s* [the difficulty in] *distinguishing between what*’*s normal and what*’*s not and what*’*s distressing and harmful and what*’*s just adolescent stuff*” (Psychiatrist).

In the absence of training in mental health, some healthcare professionals described their difficulty in determining whether young people are affected by difficult life circumstances or if they have a diagnosable mental health issue.

“*Because a lot of people have such hard*, *difficult*, *tough lives*, *I would be less likely to say that they are suffering from depression. If their brother was killed*, *their partner was just put in jail for the next five years. I will say that of course they are going to get anxious and depressed. It is not really a medical thing. It is a two*-*edged sword. There might be a higher level of awareness but also there is a certain degree of inevitability and saying this is part of the territory*” (GP).

### Identification – Enablers

#### ‘Importance of outreach work’

Healthcare professionals identified outreach work as a key enabler to identification, particularly for young people with substance use problems who are homeless. Through initial identification on the street, healthcare professionals were able to provide opportunities for young people to interact with services at a pace that is suitable for them.

“*The first point of contact might be on the street. You might see somebody begging on the street and they are an obvious opiate user*, *so*…*from street work you can offer needle exchange*, *from needle exchange you can offer Methadone*, *from Methadone you can offer stability*, *and then we can take it from there*” (Outreach Worker).

#### ‘Activity-based engagement’

The majority of healthcare professionals commended activity based programmes (e.g. sports facilities, youth clubs, youth cafes etc.) for being the most effective way of identifying mental and substance use problems among young people, where trusting relationships between healthcare professionals and young people may develop in a relaxed setting and thus provide a forum for the young person to communicate any difficulties they might be experiencing.

“*I suppose you would call the activity the carrot that they are coming to do something that they like and they are going to build the relationship through you with that. If they need support*, *in anything else*, *at least they know you and they can come to you and talk about it. You have built up the relationship in a positive way through something positive that they like to do*” (Youth Worker).

#### ‘Promoting mental health and drug awareness’

Promoting awareness and educating young people about mental health and drug use and the services that are available was identified as a priority among participants. Some of the healthcare professionals noted the importance of promoting mental health and drug awareness programmes in schools to enable early identification and remove the stigma associated with mental health problems.

“*I think one way we can overcome that* [stigma] *is by creating funky*, *cool programmes in school about being well and minding your health. Life Skill programmes*, *we all go through ups and downs and it is about coping. So introducing much earlier this notion that challenges come in life*, *our mental health gets challenged at all stages*, *this kind of approach. And*, *oh by the way*, *if you are having problems here is a signpost for you about what you need to do*” (Clinical Psychologist).

#### ‘The importance of experience, training, formal assessment and building relationships’

The importance of being experienced and having specific training in youth mental and substance use, in addition to using tools that enable formal assessment and building a trusting relationship with young people were also prioritised by healthcare professionals as key enablers to identification.

“*With emotional mental health it is not always as obvious I suppose except to the most experienced youth workers. It takes experience to recognise and identify that*” (Youth Worker).

“*I would also use formalised standards and formalised questionnaires as a means of backing up my clinical opinion as to the level of distress*” (Clinical Psychologist).

“*The employees that work in these services are so personable*, *that the young people have a tendency to attend*” (Youth Project Coordinator).

### Theme 2: Treatment – Barriers

#### ‘Limited funding’

The majority of participants stressed the negative impact of “government setbacks”, “tightly managed budgets”, staff shortages, lengthy waiting lists, bed shortages and limited resources as major barriers to offering effective treatment. As a result of the financial barriers opportunities for the healthcare professionals in the current study to replicate effective youth programmes in other countries or provide school based training programmes have been lost.

“*We have about half the staff that we are supposed to have. There are lots of things that we would like to be able to do but we are not able to do. If we had more staff for example the one thing that would be good to do is*, *in Australia they do these programmes in schools. They do a CBT programme which has been shown to reduce the number of young people who develop anxiety disorders. It would be very easy to do but we absolutely wouldn*’*t have the time to do it*” (Child Psychiatrist).

#### ‘Crisis intervention versus early intervention’

Optimum use of scarce treatment resources, especially the perceived tension between crisis intervention and early intervention, was highlighted as a priority issue. While the importance of early intervention was recognised, many healthcare professionals expressed concerns about using scarce resources for young people with less severe (rather than more severe and debilitating) problems. Promoting access to community-based psychosocial interventions was highlighted as a key mechanism to reduce workload of specialist psychiatry services.

“*If we dilute our service*…*and deal with a number of people with difficulties in adjusting to life situations*, *you dilute your service to the point where you would no longer be able to give a proper service to people with severe and enduring mental illness*” (Social Worker).

“*If there was a better provision of talking therapies* , *that would be great and that*’*s suitable for most people who would be presenting to GPs with mild to moderate depression or anxiety disorders*, [they] *can probably do just as well with a talking therapy as with any drug therapy*” (Adult Psychiatrist).

### Treatment – Enablers

#### ‘Inter-agency collaboration’

Some healthcare professionals felt that more inter-agency communication and collaboration with other healthcare professionals in different agencies, (especially between addiction services and mental health services) would benefit them in addressing the needs of young people (who might have interacted with multiple services). As the healthcare professional in the quote that follows suggests adopting an inter-agency approach where the focus is client-centred rather than being about professional competitiveness, would facilitate more effective treatment for young people with both mental and substance use disorders.

“*I think it is not only feasible*, *but I think it is imperative that they do work* [*together*], *for the good of the client*…*I don*’*t see why agencies that*, *for the most part*, *are populated by people who have got to third level education*, *and have had access to educational facilities*, *that their clients will never get near it for the most part*, *why they can*’*t put their intelligent heads together*, *and put their differences aside and work for the common good*” (Addiction Counsellor, Limerick).

Another healthcare professional commented on the benefits (particularly the positive impact of inter-agency links for young people) of having a collaborative relationship in her local area between two different healthcare sectors in the community.

“*I think that*’*s one good thing about the unity of services. Certainly I notice that the GPs are very supportive if we are running programmes in the community*, *they*’*re more than willing to put up things on their* [noticeboard] *and that shows that the system is all working together and not all working independently of one another and we are supporting them*, *they are supporting us and that in turn has an impact on the clients*” (Social Worker).

#### ‘Training in youth mental health and substance use’

Some of the participants felt “unskilled” in the area of mental health particularly GPs and the importance of receiving further training for GPs in youth mental health and addiction was suggested as a key enabler.

“*I feel very unskilled when it comes to dealing with youth mental health issues*” (GP).

*GPs are given really no training in this at all*, *anywhere along the line*, *not in college*; *maybe now in the GP schemes but when I did the GP schemes there was nothing. I think it is a big ask. If you are going to ask GPs* [to address these issues] *then you have got to train them*” (GP).

Healthcare professionals from the mental health services echoed the need for further training particularly for GPs to avoid inappropriate referrals to their service.

“*GPs need to be up skilled in the type of assessment they do for mental health problems. Some of the referrals are good*, *and some are terrible that we get in. So*, *initially the GPs probably should be up skilled a little bit in doing a fuller assessment of the nature of the problems*” (Social Worker).

### Theme 3: On-going Engagement – Barriers

#### ‘External pressure to engage in treatment’

Healthcare professionals recalled their struggle to work with young people who were not intrinsically motivated to engage with them during the treatment process. In the majority of cases healthcare professionals noted that young people attend services as a result of pressure from external factors e.g. parents, social workers, probation officers etc.

“*This guy doesn*’*t want to be in there. He has not come in specifically*; *I want to address my ADHD*, *I want to address my substance use and I want to address the fact that I am involved in anti*-*social behaviour. He was given a letter saying that you must attend*, *you must*…*That is not going to work. You do what you can with it*” (Drug Worker).

#### ‘Unwillingness to attend counselling’

Other healthcare providers struggled to motivate young people to attend counselling for various reasons e.g. counselling being perceived as a ‘middle-class’ intervention, the time commitment associated with engaging in counselling and a fear of bringing up painful memories. Some of the healthcare professionals found that young people wanted a “quick fix”/ immediate solution in the form of a tablet rather than to engage / interact with healthcare professionals in any form of counselling.

“*A lot of people come in just wanting tablets*, *particularly Benzodiazepines. And we say* “*let*’*s do some relaxation or anxiety management techniques*” *and they say* “*no*, *I just want a tablet*.” *So I think there is a culture of* “*I want it straight away*, *and I want you to make me better*” *without people taking responsibility for their own health*, *and doing what needs to be done. So that might be a barrier as well*” (Social Worker).

#### ‘Transition from child to adult services’

For healthcare professionals trying to ease the transition for young people from child to adult mental health services at 18 years was also identified as a barrier to on-going engagement, especially as many young people may have developed a trusting relationship with a member of the clinical team which they are reluctant to end.

“*It*’*s the wrong time for there to be a transition in care because transitions in care are unsatisfactory. People get lost you know and relationships get broken up*” (Psychiatrist).

### On-going Engagement – Enablers

#### ‘Intrinsic motivation’

The majority of healthcare providers emphasised the need for young people to be intrinsically motivated to attend services and continue with their treatment. One of the participants highlighted the benefits of motivational interviewing with young people to increase confidence and address any “slips” during treatment engagement.

“*People grow in confidence by being respected in whatever effort they make and*....*I would do an awful lot of motivational interviewing. I would really*....*be attentive to the positive and just be aware of the negative. And if they have a slip*, *so what*? *Of course they slip but the important thing is why*, *I would explore the reasons. Maybe they had a row. Somebody said something nasty to them*, *or they perceived somebody said something nasty to them. We would kind of explore that*” (Addiction Counsellor).

#### ‘Continued opportunities for engagement’

Healthcare professionals also advocated the importance of providing continued opportunities for young people to engage with services given the infrequent / relaxed approach that some young people have when it comes to keeping appointments. One of the participants stressed the importance of “being flexible” in their “attitudes” as healthcare professionals towards understanding how young people engage with services.

“*Sometimes being that facilitator and opening up that avenue of support even though it is not taken up. It might be eighteen months*, *two years or as recently as four years down the road. We will open the file and we will leave it there. Very often you will hear people ringing to make an appointment and two or three years later they turn up. That is progress. It is long*, *it is drawn out and it demands patience. It depends on an ability to be flexible in terms of our own attitudes*” (Counselling Psychologist).

#### ‘Personal achievement goals’

One of the healthcare professionals stressed the importance of building on and commending the young person’s personal achievements as a key enabler to on-going engagement. Additional strategies included connecting them with other people in the recovery programme, encouraging return to school, leisure activities, return to work initiatives etc.

“*Some would say* “*I never thought I would do three days without cannabis*”. *That would be a major* (*achievement for them*), *and every day you build on that and try to reconnect them with other people who are in recovery as well. And*, *really applaud each little step they make in the right direction. Because*....*anybody who really stops*, *I would so affirm every effort they make. And to re*-*engage maybe in school*…*other activities*” (Addiction Counsellor).

## Discussion

### Key findings

We identified three themes (identification, treatment and engagement) and associated barriers and enablers, in respect of interventions to promote screening and treatment for mental and substance use disorders among young people in primary care. Healthcare professionals felt restricted in the identification of young people with mental and substance use disorders due to the need to prioritise emergency / crisis cases over young people with less severe problems, consent and confidentiality issues and fear of the future implications that might be associated with a formal diagnosis. In the absence of sufficient training in mental health, distinguishing between the impact of difficult life circumstance and the symptoms of a mental health problem proved to be a major barrier to identification. However identification of youth mental and substance use disorders were facilitated by outreach work and activity based programmes where healthcare professionals had the opportunity to interact with young people in a less formalised setting and therefore build positive relationships. Specific training in mental health and the use of formalised assessment tools were also identified as key enablers to identification.

Barriers to treatment were mainly due to financial cuts, which hindered healthcare professionals in their efforts to offer the level of treatment that they would like to. Healthcare professionals working in the mental health services felt that their services should only be utilised for young people with severe problems and in most cases community based services would be sufficient for young people experiencing mild psychological difficulties. Enablers to treatment included an inter-agency approach between services, further training particularly for GPs who felt “unskilled” in the area of mental health. Barriers to on-going engagement included external pressure on young people to attend services, young people’s reluctance to engage in counselling, and moving young people to adult mental health services. On-going engagement was facilitated by helping young people to be intrinsically motivated during treatment, providing continued opportunities to engage with services and building on the young person’s personal achievement goals during the recovery process.

### Strengths and limitations of the study

Our qualitative approach allowed us to develop an in-depth understanding of the difficulties encountered in treating young people with mental health and substance use difficulties. Our sampling methods are likely to have biased participants towards those practitioners more engaged with youth mental health. The applicability of our findings to practitioners who are relatively less engaged with this issue should be the focus of future research. Incorporating a broad range of stakeholders from diverse clinical settings was beneficial in terms of reflecting the various sites where young people seek help for mental and substance use disorders. However, differences and similarities in opinion between professional disciplines in regards to the challenges experienced warrants further analysis. The extreme variance in interview length with healthcare providers e.g. 16–120 minutes is another limitation. All of the participants were interviewed in their work place, thus in some cases the interview duration was dependent on external factors related to the participant’s work environment. An additional limitation to this study was the inability to remove researcher bias as four of the authors (DL, ES, CA and WC) analysed the data.

### Comparison with existing literature

Our findings regarding enablers of identification, treatment and further engagement compare to those previously documented, including outreach, education and awareness, intrinsic motivation, positive relationships with healthcare professionals, familiarity with a clinic or practice [2,42]–[45]. Our findings also highlight the value of service configuration, especially inter-agency collaboration [2,46,47], accessibility [48], transition services that ensure on-going care for young people [30] and further service integration [49].

Youth mental health was clearly a priority for this study’s participants; and in keeping with the Social Determinants of Health, social deprivation and social cohesion are clearly important factors in the origins, treatment and outcomes of this problem [50]. In keeping with the ‘Chronic Care Model’, the value of healthcare organization, system redesign and community resources were highlighted, though it is worth noting that other elements of this model (self-management support, clinical information systems, decision support) were not [37].

### Implications for further research and clinical practice

Given the considerable contact many young people have with primary care and its longitudinal nature [51] it is vital that healthcare professionals in primary care are equipped to identify such problems accurately and early. Thus a strong focus on youth mental health within undergraduate and postgraduate health programmes is vital, in addition to further training for existing professionals in the field [47,52]. Providing a safe and supportive environment in which a young person can initiate a conversation on mental health, ensuring healthcare professionals are confident in this conversation and have appropriate services to which they can refer more challenging cases, are key to early intervention.

Complex interventions to support formal identification and treatment of mental and substance use disorders can enhance healthcare professionals’ knowledge, skill, competency and practice [53]. Education, supported by resources such as the ‘Australian Adolescent Health GP Resource Kit’ is likely to be a central component of such interventions [54], however the implications of using this resource in a different culture cannot be ignored.

Further research to enhance our understanding of this issue would include epidemiological studies and qualitative accounts of young people’s experience. Greater understanding will then aid the development and evaluation of complex interventions that promote early intervention in deprived areas. It seems likely that interventions would be based on promoting mental health awareness in the community, education of practitioners, improved access to psychological treatments, and greater access to specialist care for those with more complex morbidity.

## Conclusions

With youth mental health considered a key agenda to be included among the global health targets [52], the need to improve how primary care engages with this population is crucial. A responsive youth mental health system would ideally moderate the emotional distress of the young person concerned and their families, while reducing the financial burden of chronic adult mental illness [55]. While primary care might be well placed to address mental and substance use disorders in young people, healthcare professionals are presented with many challenges in fulfilling this task. Limited financial resources, lack of training and available mental health services for referral cannot be ignored. Structural changes are necessary across the healthcare spectrum if healthcare professionals are to succeed in providing early intervention for young people with mental and substance use issues. Complex strategies that promote identification, treatment and ongoing engagement are important elements to such a system, and would need to address areas such as outreach, awareness, intrinsic motivation, positive relationships with healthcare professionals, familiarity with a clinic or practice, and service configuration.

## Appendix 1. Topic Guide – Healthcare Professionals

Demography/Descriptive Data

1) How long have you been in your current profession?

2) What kind of training have you had in youth mental health?

3) How do you usually become aware of young people who might have a mental health or substance use disorder?

4) What proportion of your time is spent working with young people with such conditions?

5) Can you tell me about your previous/current practice of screening/early intervention for mental distress and/or substance use amongst young people?

Experience of mental and substance use disorders among young people

1) How are young people’s needs identified?

2) What are the main challenges in regards to meeting the needs of young people with respect to:

a) treatment engagement?

a) treatment sustainment?

a) need identification?

a) resources available?

a) differences between adults and young people?

3) Are there additional supports / community resources available outside of this service for young people?

a) If so…can you tell me more about them?

4) How would you improve your service with respect to:

a) access to services for young people?

5) What is your view on the inclusion of parents/guardians in a young person’s treatment for mental/substance use difficulties?

*Attitude towards screening*/*early intervention*

1) Do you think it would be feasible to have screening in your service?

2) What are the main factors that facilitate screening/early intervention for mental distress/substance use in young people?

3) What are the main barriers that prevent screening/early intervention for mental distress/substance use in young people?

4) If the child of a friend of yours had a mental health or substance use difficulty, what would you advise them to do in the first instance?

5) If you have a young person presenting with both mental and substance use difficulties what kind of treatment options are available to them?

6) Could you tell me briefly about a young person that you cared for that resulted in a positive outcome? What was the condition? How did you help? Why was the outcome so good?

7) Are there any other comments you would like to make?

## Competing interests

The authors declare that they have no competing interest.

## Author’s contributions

WC conceived the study and wrote the proposal, obtained research funding / ethics approval and established project steering group. DL, ES and CA collected the data in collaboration with WC, DM, BG, EK, FM, ROC, VK, BS, EOD, RD, BD, LL, PCM and ED. DL, ES and CA analysed the data in collaboration with WC. WC, DL, ES, CA, DM, BG, EK, FM, ROC, VK, PR, LS, TOT, RK, EOD, ER, and BS drafted the manuscript. All authors read and approved the final manuscript.

## Pre-publication history

The pre-publication history for this paper can be accessed here:

http://www.biomedcentral.com/1471-2296/14/194/prepub
